# Blockage of Galectin-receptor Interactions by α-lactose Exacerbates *Plasmodium berghei*-induced Pulmonary Immunopathology

**DOI:** 10.1038/srep32024

**Published:** 2016-08-24

**Authors:** Jinfeng Liu, Shiguang Huang, Xin-zhuan Su, Jianping Song, Fangli Lu

**Affiliations:** 1 Department of Parasitology, Zhongshan School of Medicine, Sun Yat-sen University, Guangzhou 510080, Guangdong, China; 2 Key Laboratory of Tropical Disease Control (Sun Yat-sen University), Ministry of Education, Guangzhou 510080, Guangdong, China; 3 School of Medicine, Jinan University, Guangzhou 510632, Guangdong, China; 4 Laboratory of Malaria and Vector Research, National Institute of Allergy and Infectious Diseases, National Institutes of Health, Bethesda, Maryland 20892, United States of America; 5 State Key Laboratory of Cellular Stress Biology, Innovation Center for Cell Signaling Network, School of Life Sciences, Xiamen University, Xiamen, Fujian 361005, China; 6 Institute of Science and Technology, Guangzhou University of Chinese Medicine, 436 Chentai Road, Baiyun District, Guangzhou 510445, Guangdong, China

## Abstract

Malaria-associated acute lung injury (ALI) is a frequent complication of severe malaria that is often caused by “excessive” immune responses. To better understand the mechanism of ALI in malaria infection, here we investigated the roles of galectin (Gal)-1, 3, 8, 9 and the receptors of Gal-9 (Tim-3, CD44, CD137, and PDI) in malaria-induced ALI. We injected alpha (α)-lactose into mice-infected with *Plasmodium berghei* ANKA (*Pb*ANKA) to block galectins and found significantly elevated total proteins in bronchoalveolar lavage fluid, higher parasitemia and tissue parasite burden, and increased numbers of CD68^+^ alveolar macrophages as well as apoptotic cells in the lungs after blockage. Additionally, mRNA levels of Gal-9, Tim-3, CD44, CD137, and PDI were significantly increased in the lungs at day 5 after infection, and the levels of CD137, IFN-α, IFN-β, IFN-γ, IL-4, and IL-10 in the lungs were also increased after α-lactose treatment. Similarly, the levels of Gal-9, Tim-3, IFN-α, IFN-β, IFN-γ, and IL-10 were all significantly increased in murine peritoneal macrophages co-cultured with *Pb*ANKA-infected red blood cells *in vitro*; but only IFN-α and IFN-β were significantly increased after α-lactose treatment. Our data indicate that Gal-9 interaction with its multiple receptors play an important role in murine malaria-associated ALI.

Malaria is still a major global health problem. Severe malaria, associated with high morbidity and mortality, is characterized by cerebral malaria, severe anemia, acidosis and hypoglycemia, pulmonary edema, and acute kidney injury^1^. Pulmonary edema, which can be caused by infection with *Plasmodium falciparum*, *P*. *vivax*, or *P*. *ovale*^2^, is featured by acute lung injury (ALI) and acute respiratory distress syndrome (ARDS) and occurs in approximately 20% of severe malaria patients^3^. ALI develops prior to the onset of cerebral malaria symptoms, and malaria-induced ALI/ARDS pathophysiology demonstrates that inflammatory-mediated increased capillary permeability or endothelial damage leads to diffuse alveolar destruction that can continue after parasite clearance^4^. It has been reported that parasite burden and CD36-mediated sequestration in the lung are primary determinants of ALI in experimental murine malaria^5^, and significantly higher rate of monocytes and macrophages are detected in placenta with falciparum malaria infections^6^. However, so far few studies have addressed the characteristics of the immunological response of macrophage infiltration during malaria-induced ALI.

Galectins, beta-galactoside-binding animal lectins, are differentially expressed by various immune cells as well as a wide range of other cell types^7^. So far, fifteen members of the galectin family have been identified in vertebrates^8^. Studies have reported that age-related susceptibility to severe malaria is associated with Gal-2 in highland Papuans^9^. Gal-3 protein is overexpressed in mice of experimental cerebral malaria (ECM) and deletion of the Gal-3 gene confers partial but significant protection from ECM in C57BL/6 mice challenged with *P*. *berghei* ANKA (*Pb*ANKA), suggesting Gal-3 can also alter the pathogenic course of ECM^10^. In addition, Gal-9, but not Gal-3, can bind to the *L*. *major*-specific polygalactosyl epitope and promote interaction between *L*. *major* and macrophages, suggesting a differential role of galectins in leishmaniasis^11^.

Studies have demonstrated that T cell Ig and mucin domain–containing molecule-3 (Tim-3) is expressed on cells of both innate and adaptive immune systems, including T cells, dendritic cells (DCs), macrophages, etc^12^. Gal-9 is identified as a Tim-3 ligand and the Gal-9/Tim-3 interaction acts as a specific inhibitor of T helper (Th)1 and Th17 immune responses^13^; however studies have shown that Gal-9 also binds to CD44^14^, CD137^15^, and protein disulfide isomerase (PDI)^16^. In addition, high-mobility group box 1 (HMGB-1) also binds to Tim-3^17^. CD44 is a transmembrane adhesion molecule that is present on a wide variety of cell types, including leukocytes and parenchymal cells, and is an important player in leukocyte trafficking^18^.

Tim-3/Gal-9 interaction plays a crucial role in immune regulation, and it mediates proinflammatory cytokine secretion in DCs and macrophages and promotes inflammation^12^. Macrophages act as the primary host cell for many intracellular pathogens. Once infected, intracytosolic pathogens induce type I IFNs (IFN-I) expression in DCs and macrophages, which enhances the susceptibility of mice to *Salmonella enterica* serovar Typhimurium (*S*. Typhimurium) infection^19^. Although the high mortality of ALI during severe malaria highlights the importance of this syndrome during malarial infection, the immunopathological mechanisms that lead to ALI/ARDS in malarial infection remain largely unknown. Currently, it is not clear what role of galectins play in malaria associated-ALI and whether manipulating galectin binding to their receptors can influence the magnitude and effectiveness of malarial immunity. The pathway involved in the induction of IFN-I by the interaction of Gal-9 and its receptors in immunopathogenesis of lung injury during malarial parasite infection have not been reported. In this study, we investigated the roles of Gal-1, Gal-3, Gal-8, Gal-9, the receptors of Gal-9 (Tim-3, CD44, CD137, and PDI), IFN-I (IFN-α and IFN-β), IFN-II (IFN-γ), IL-4, IL-10, and their interactions with macrophages during *Pb*ANKA infection with or without blockage of galectins by α-lactose *in vivo* and *in vitro*. We demonstrated that interactions of Gal-9 and its receptors (Tim-3, CD44, CD137, and PDI) play a role in the development of ALI in *Pb*ANKA-infected mouse model, providing an important mechanism for severe malaria.

## Results

### Blockage of galectins by α-lactose increased bronchoalveolar lavage fluid (BALF) total protein, parasitemia/tissue parasite burden, and host mortality

**Figure 1:**
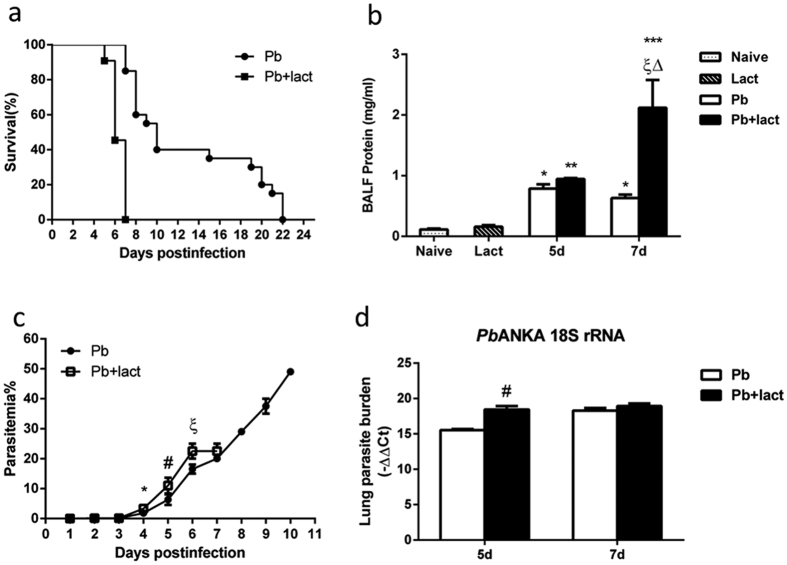
Parasitemia, survival rate, total protein in the BALFs, and lung parasite burden in *Pb*ANKA-infected mice with or without α-lactose treatment. (**a**) Survival rate. Malarial mice (n = 20) died between days 7 and 20 p.i. Malarial mice treated with α-lactose (n = 11) died between days 5 and 7 p.i. (**b**) Total protein levels in BALFs were measured using BCA Protein Assay. *P < 0.05; **P < 0.01 and ***P < 0.001, malarial mice on 5 days p.i., malarial mice treated with α-lactose on 5 days p.i., malarial mice on 7 days p.i., and malarial mice treated with α-lactose on 7 days p.i. vs. naive group; ξ, *P* < 0.05, malarial mice treated with α-lactose on 7 days p.i. vs. malarial mice at 7 days p.i.; Δ, *P* < 0.05, malarial mice treated with α-lactose on 7 days p.i. vs. malarial mice treated with α-lactose on 5 days p.i. There were four mice per group, and data are representative of those from two experiments. (**c**) Time course of parasitemia of malarial mice and malarial mice treated with α-lactose. Parasitemias are shown as mean ± SD. *P < 0.05, malarial mice treated with α-lactose on 4 days p.i. vs. malarial mice on 4 days p.i.; ^#^*P* < 0.05, malarial mice treated with α-lactose on 5 days p.i. vs. malarial mice on 5 days p.i.; ξ, *P* < 0.05, malarial mice treated with α-lactose on 6 days p.i. vs. malarial mice on 6 days p.i. There were four mice per group, and data are representative of those from two experiments. (**d**) Parasite burden estimated using mRNA level of *Pb*ANKA 18S rRNA in the lungs measured by qRT-PCR. Values are means from triplicate measurements, and data are shown as −∆∆Ct values. ^#^*P* < 0.05, malarial mice treated with α-lactose on 5 days p.i. vs. malarial mice on 5 days p.i. There were four mice per group, and data are representative of those from two experiments.

The symptoms and parasitemia of mice were monitored daily after *Pb*ANKA injection. *Pb*ANKA-infected mice (malarial mice) died between 5 and 22 days post infection (p.i.), however, malarial mice treated with α-lactose had 100% mortality by day 7 p.i. (Fig. 1a). To evaluate the lung damage after *Pb*ANKA infection, bronchoalveolar lavage (BAL) was performed in mice on days 5 and 7 p.i., and the BALFs were examined for protein content. As shown in Fig. 1b, compared with uninfected controls, there were significantly increased total protein levels in the BALFs in malarial mice on days 5 and 7 p.i. (*P* < 0.05), and significantly increased total protein levels in malarial mice treated with α-lactose in comparison of malarial mice on day 7 p.i. (*P* < 0.05). Significantly higher level of total protein in the BALFs was also observed in malarial mice treated with α-lactose on day 7 p.i. (*P* < 0.05) in comparison of that on day 5 p.i., indicating a disruption of the alveolar-capillary membrane barrier as a result of *Pb*ANKA infection. Parasitemia in malarial mice treated with α-lactose was also significantly higher than that of malarial mice on days 4, 5, and 6 p.i. (*P* < 0.05) (Fig. 1c). However, there was no significant difference of parasitemia on day 7 p.i. (*P* > 0.05) between the two groups. In addition, the parasite burdens in lung tissues were measured using qRT-PCR; there was significantly increased parasite burden on day 5 p.i. (*P* < 0.05) in malarial mice treated with α-lactose in comparison of malarial mice; however, no significant difference of parasite burden was detected on day 7 p.i. (*P* > 0.05) between the two groups (Fig. 1d).

### Blockage of galectins by α-lactose promoted lung pathology

**Figure 2:**
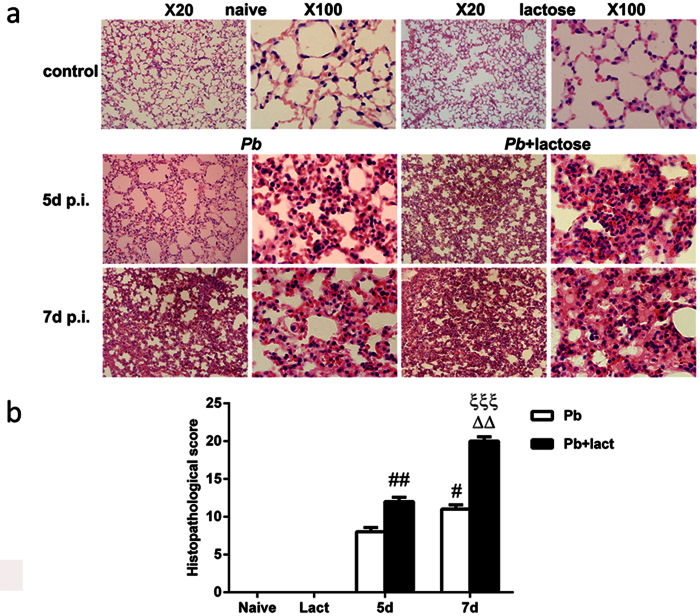
Histopathology of lungs in *Pb*ANKA-infected mice with or without α-lactose treatment. (**a**) Histopathological changes in the lungs. H&E stain. (**b**) Histopathological score analysis of the lungs. Data are represented as mean ± SD. Significant differences between groups were analyzed using Wilcoxon rank sum test. ^#^*P* < 0.05 and ^##^*P* < 0.01, malarial mice treated with α-lactose on 5 days p.i. and malarial mice on 7 days p.i. vs. malarial mice on 5 days p.i.; ΔΔ, *P* < 0.01, malarial mice treated with α-lactose on 7 days p.i. vs. malarial mice treated with α-lactose on 5 days p.i.; ξξξ, *P* < 0.001, malarial mice treated with α-lactose on 7 days p.i. vs. malarial mice on 7 days p.i. There were four mice per group, and data are representative of those from two experiments.

The lung tissues of mice were examined histologically. As shown in Fig. 2a, tissue sections of lungs from uninfected mice and uninfected mice treated with α-lactose had no obvious morphological or structural abnormalities. However, histopathological analysis in the lung tissues revealed abundant *Pb*ANKA-infected red blood cells (iRBC) in the interalveolar spaces of the lungs from malarial mice and malarial mice treated with α-lactose. Increased cell infiltration including lymphocytes, mononuclear cells, and neutrophils was observed in the lungs of malarial mice treated with α-lactose on day 5 p.i., leading to severe inflammation and tissue damage on day 7 p.i. in the α-lactose treated malarial mice (Fig. 2b). Semi-quantitative lung inflammation scores based on pathological changes of the lung tissues showed significantly elevated scores in the lungs of α-lactose-treated malarial mice on days 5 (*P* < 0.01) and 7 (*P* < 0.001) p.i.

### Blockage of galectins by α-lactose increased the number of CD68^+^ macrophages in the lungs

**Figure 3:**
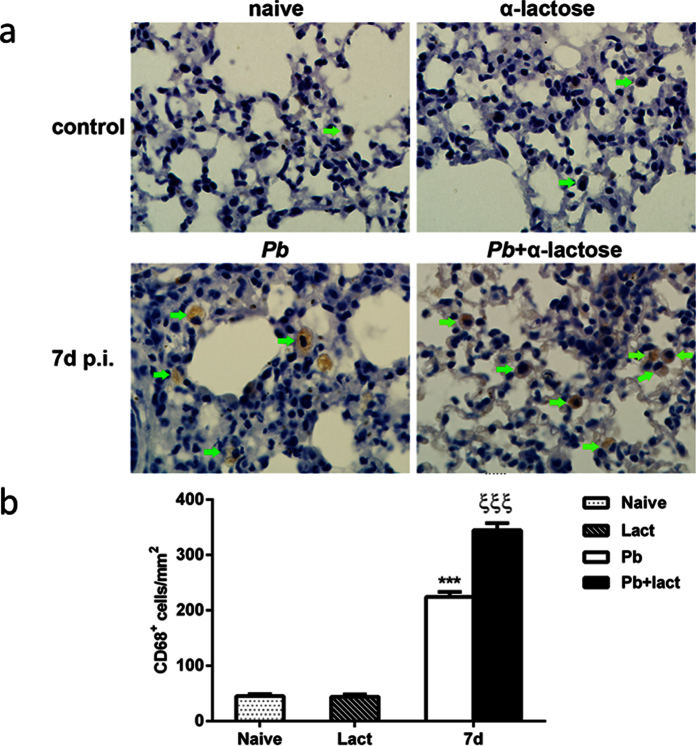
Immunohistochemical staining for CD68^+^ macrophages in the lungs of *Pb*ANKA-infected mice with or without α-lactose treatment. (**a**) Immunohistochemical staining of CD68^+^ macrophages (green arrows) in the lungs of uninfected mice, uninfected mice treated with α-lactose, malarial mice, and malarial mice treated with α-lactose on days 7 p.i. Original magnification ×1,000. (**b**) Morphometric analysis of lung tissues. Shown are CD68^+^ macrophages per square millimeter. Data are presented as means ± SD; experiments were performed with three mice per group. ***P < 0.001, malarial mice on 7 days p.i. and malarial mice treated with α-lactose on 7 days p.i. vs. naive group; ξξξ, *P* < 0.001, malarial mice treated with α-lactose on 7 days p.i. vs. malarial mice on 7 days p.i.

As shown in Fig. 3a, there were few CD68^+^ macrophages (brown color) in the lung tissues of uninfected controls. However, the architecture of lung tissues was massively distorted, and large number of macrophages was dispersed in the interstitial and alveolar spaces in the lung sections of malarial mice on day 7 p.i. Compared with uninfected controls, there were significantly higher numbers of CD68^+^ macrophages in the lungs of *Pb*ANKA-infected mice on day 7 p.i. (*P* < 0.001) and significantly more CD68^+^ macrophages in the lung tissues of malarial mice treated with α-lactose than those of malarial mice on day 7 p.i. (*P* < 0.001) (Fig. 3b).

### Blockage of galectins by α-lactose modulated apoptosis in the lungs

**Figure 4:**
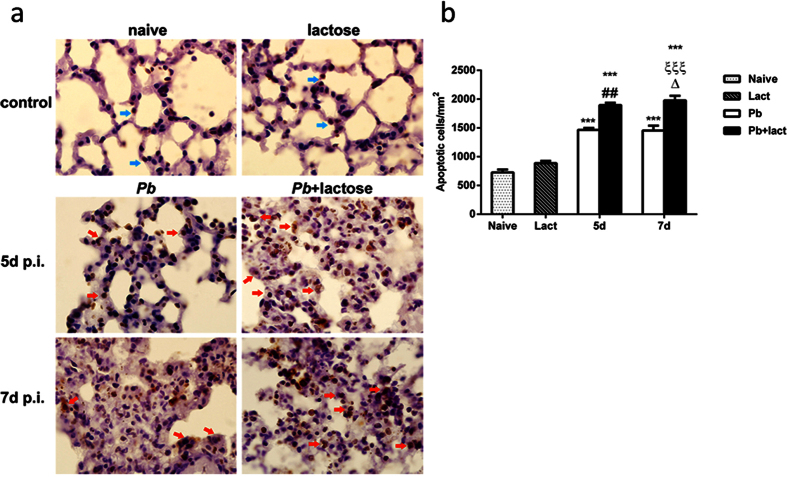
TUNEL staining of the lung tissues of *Pb*ANKA-infected mice with or without α-lactose treatment. Shown are representative lung sections from TUNEL staining. (**a**) Apoptotic lymphocytes (blue arrows) in the lungs of naive mice and mice treated with α-lactose, and apoptotic mononuclear cells (red arrows) in the lungs of malarial mice and malarial mice treated with α-lactose on 5 and 7 days p.i. Original magnification ×1,000. (**b**) Morphometric analysis of apoptotic cells in the lung tissues. Shown are apoptotic cells per square millimeter. Data are presented as means ± SD; experiments were performed with three mice per group. ***P < 0.001, malarial mice on 5 days p.i., malarial mice treated with α-lactose at 5 days p.i., malarial mice on 7 days p.i., and malarial mice treated with α-lactose on 7 days p.i. vs. naive group; ^##^*P* < 0.01, malarial mice treated with α-lactose on 5 days p.i. vs. malarial mice on 5 days p.i.; Δ, *P* < 0.05, malarial mice treated with α-lactose on 7 days p.i. vs. malarial mice treated with α-lactose on 5 days p.i.; ξξξ, *P* < 0.001, malarial mice treated with α-lactose on 7 days p.i. vs. malarial mice on 7 days p.i.

TUNEL assay was used to evaluate the effects of blockage of galectins by α-lactose on apoptosis in the lungs. Apoptotic cells including lymphocytes, mononuclear cells, and neutrophils in the lungs in both malarial mice and malarial mice treated with α-lactose were observed on days 5 and 7 p.i., while only a few apoptotic cells were observed in the lung tissues of uninfected controls. More apoptotic mononuclear cells in the lungs of malarial mice treated with α-lactose than those of malarial mice were observed on days 5 and 7 p.i. (Fig. 4a). Quantitative analysis of TUNEL positive cells in the lung tissues showed that the numbers of apoptotic cells in malarial mice treated with α-lactose were significantly higher than those in malarial mice on days 5 (*P* < 0.01) and 7 (*P* < 0.001) p.i. Malarial mice treated with α-lactose had more apoptotic cells in the lungs on day 7 p.i. than those on day 5 p.i. (*P* < 0.05) (Fig. 4b), likely due to higher level of inflammatory response.

### Gal-9 and Tim-3 expressions on macrophages from BALFs and in the lungs

**Figure 5:**
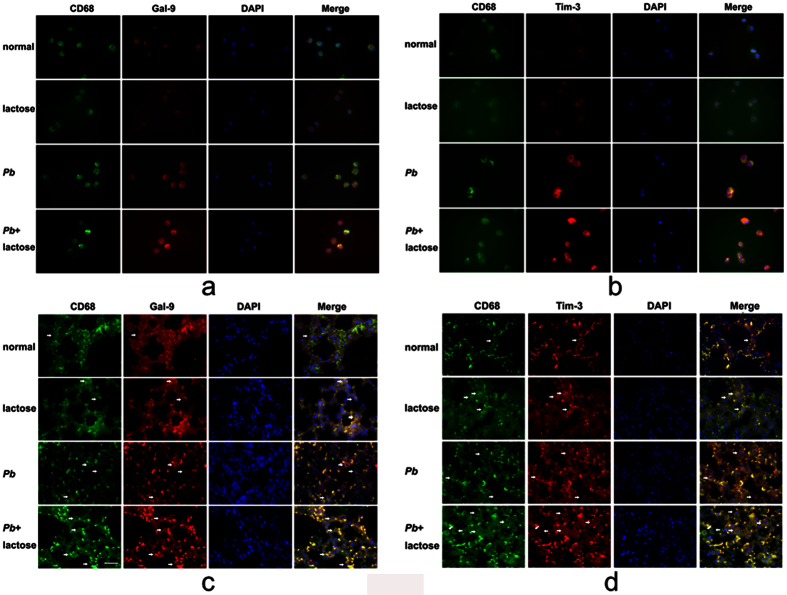
Immunofluorescence staining for CD68^+^-Gal-9^+^ and CD68^+^-Tim-3^+^macrophages from the BALFs and in the lung tissues of *Pb*ANKA-infected mice with or without α-lactose treatment. Double immunofluorescence showing co-localization of Gal-9 or Tim-3 on CD68^+^ macrophages (white arrows). (**a**) CD68^+^-Gal-9^+^ macrophages from the BALFs of malarial mice and malarial mice treated with α-lactose on 7 days p.i. in comparison with naive and α-lactose-control under the same exposure conditions. (**b**) CD68^+^-Tim-3^+^ cells from the BALFs of malarial mice and malarial mice treated with α-lactose on 7 days p.i. in comparison with naive and α-lactose-control under the same exposure time. (**c**) CD68^+^-Gal-9^+^ macrophages in the lung tissues of malarial mice and malarial mice treated with α-lactose on 7 days p.i. (**d**) CD68^+^-Tim-3^+^ macrophages in the lung tissues of malarial mice and malarial mice treated with α-lactose on 7 days p.i. in comparison with naive and α-lactose-control. Original magnification ×1,000.

We next investigated the expressions for Gal-9 and Tim-3 on CD68^+^ macrophages from BALFs using immunofluorescence assay. The results showed that both Gal-9 (Fig. 5a) and its receptor Tim-3 (Fig. 5b) were expressed on CD68^+^ macrophages in this model. Compared with controls (uninfected and untreated/uninfected and α-lactose-treated), under the same exposure conditions, stronger Gal-9 and Tim-3 signals were observed on BALF CD68^+^ macrophages from both malarial mice and malarial mice treated with α-lactose. Similarly, both Gal-9 (Fig. 5c) and Tim-3 (Fig. 5d) were expressed on the CD68^+^ macrophages in the lung tissues of all the groups. Compared with normal control (uninfected and untreated), more CD68^+^-Gal-9^+^ and CD68^+^-Tim-3^+^ positive cells were observed in α-lactose-control, malarial mice, and malarial mice treated with α-lactose (except Tim-3 in uninfected and α-lactose-treated). These results suggest the Gal-9 and Tim-3 play a role in the inflammatory response in the lung infected with malarial parasite.

### Blockage of Gal-9/Tim-3 pathway led to increased IFN-I production in murine peritoneal macrophages co-cultured with *Pb*ANKA-iRBCs *in vitro* and *in vivo*

**Figure 6:**
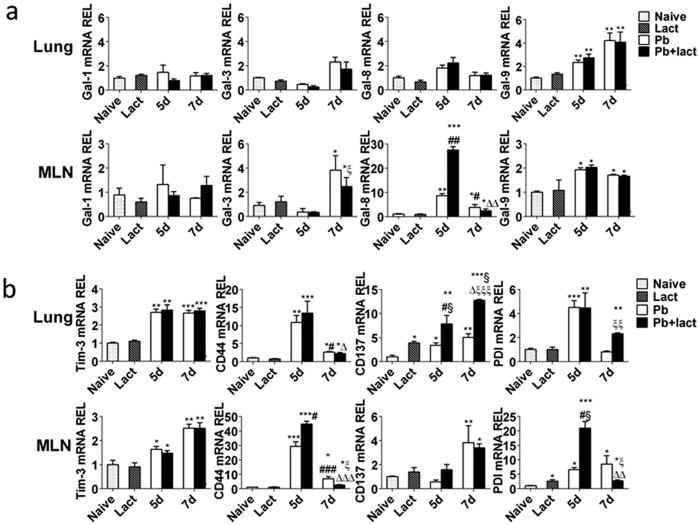
mRNA levels of galectins and the receptors of Gal-9 in the lung and MLN tissues of *Pb*ANKA-infected mice with or without α-lactose treatment estimated using qRT-PCR. (**a**) mRNA levels of Gal-1, Gal-3, Gal-8, and Gal-9; (**b**) mRNA levels of Tim-3, CD44, CD137, and PDI. Values are means from triplicate measurements, and data are presented as means ± SD; two independent experiments were performed with four mice per group. *P < 0.05; **P < 0.01; and ***P < 0.001, α-lactose-control, malarial mice on 5 days p.i., malarial mice treated with α-lactose on 5 days p.i., malarial mice on 7 days p.i., and malarial mice treated with α-lactose on 7 days p.i. vs. naive group; §, *P* < 0.05, malarial mice treated with α-lactose on 5 days p.i. and malarial mice treated with α-lactose on 7 days p.i. vs. α-lactose-control; ^#^*P* < 0.05; ^##^*P* < 0.01; and ^###^*P* < 0.001, malarial mice treated with α-lactose on 5 days p.i. and malarial mice at 7 days p.i. vs. malarial mice on 5 days p.i.; Δ, *P* < 0.05; ΔΔ, *P* < 0.01; and ΔΔΔ, *P* < 0.001, malarial mice treated with α-lactose on 7 days p.i. vs. malarial treated with α-lactose on 5 days p.i.; ξ, *P* < 0.05; ξξ, *P* < 0.01; and ξξξ, *P* < 0.001, malarial mice treated with α-lactose on 7 days p.i. vs. malarial mice on 7 days p.i. REL: relative expression level.

**Figure 7:**
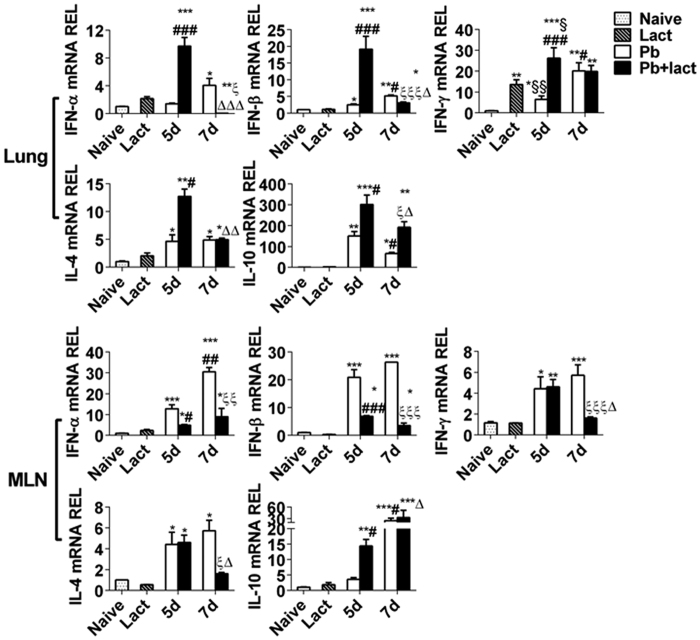
mRNA levels of IFN-α, IFN-β, IFN-γ, IL-4, and IL-10 in the lung and MLN tissues of *Pb*ANKA-infected mice with or without α-lactose treatment estimated using qRT-PCR. Values are means from triplicate measurements, and data are presented as means ± SD; two independent experiments were performed with four mice per group. *P < 0.05; **P < 0.01; and ***P < 0.001, α-lactose-control, malarial mice on 5 days p.i., malarial mice treated with α-lactose on 5 days p.i., malarial mice on 7 days p.i., and malarial mice treated with α-lactose on 7 days p.i. vs. naive group; §, *P* < 0.05 and §§, *P* < 0.01, malarial mice on 5 days p.i. and malarial mice treated with α-lactose on 5 days p.i. vs. α-lactose-control; ^#^*P* < 0.05; ^##^*P* < 0.01; and ^###^*P* < 0.001, malarial mice treated with α-lactose on 5 days p.i. and malarial mice at 7 days p.i. vs. malarial mice on 5 days p.i.; Δ, *P* < 0.05; ΔΔ, *P* < 0.01; and ΔΔΔ, *P* < 0.001, malarial mice treated with α-lactose on 7 days p.i. vs. malarial treated with α-lactose on 5 days p.i.; ξ, *P* < 0.05; ξξ, *P* < 0.01; and ξξξ, *P* < 0.001, malarial mice treated with α-lactose on 7 days p.i. vs. malarial mice on 7 days p.i. REL: relative expression level.

We investigated the expression of CD44, CD137, and PDI in the lung after infection with the parasite on days 5 and 7 p.i.; because it has been shown that these molecules can also act as receptors for Gal-9^14^,^15^,^16^. Except Gal-9 (*P* < 0.05), other galectins (Gal-1, Gal-3, and Gal-8) did not respond significantly to parasite infection in the lungs of *Pb*ANKA-infected mice with or without α-lactose treatment; however, Gal-3, Gal-8, and Gal-9 were all significantly increased in the MLNs of *Pb*ANKA-infected mice with or without α-lactose treatment (Fig. 6a). Interestingly, the expressions of Gal-9 receptors (Tim-3, CD44, CD137, and PDI) were all significantly increased in both the lung and MLNs after infection; however, significantly increased expressions of CD137 and PDI in the lungs and CD44 and PDI in the MLNs were detected on day 5 and/or day 7 after α-lactose treatment (Fig. 6b). These results suggest that in addition to Tim-3, other Gal-9 receptors (CD44, CD137, and PDI) may also contribute to the disease phenotypes *in vivo*. Although no significant change in expression of Tim-3 was observed after α-lactose treatment, the effects of blockage of Gal-9 and Tim-3 engagement could still significantly influence the downstream genes. We also measured the mRNA levels of IFN-α, IFN-β, IFN-γ, IL-4, and IL-10 after infection with or without α-lactose treatment on days 5 and 7 p.i. As shown in Fig. 7, in the lungs, significantly higher mRNA levels of all the genes were observed on day 5 p.i. after α-lactose treatment; in the MLNs, only IL-10 was significantly increased on day 5 p.i. after α-lactose treatment, whereas IFN-α, IFN-β, IFN-γ, and IL-4 were significantly decreased on day 5 p.i. and/or on day 7 p.i. after α-lactose treatment.

**Figure 8:**
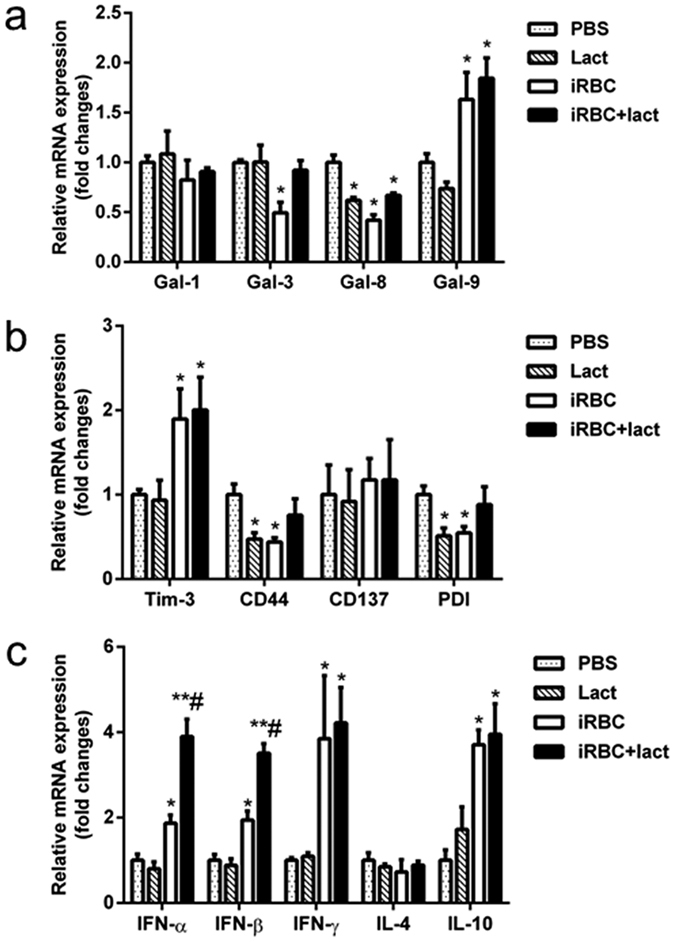
mRNA levels of galectins, the receptors of Gal-9, and cytokines in peritoneal macrophages co-cultured with *Pb*ANKA-iRBCs. (**a**) mRNA levels of Gal-1, Gal-3, Gal-8, and Gal-9; (**b**) mRNA levels of Tim-3, CD44, CD137, and PDI; and (**c**) mRNA levels of IFN-α, IFN-β, IFN-γ, IL-4, and IL-10. Values are means from triplicate measurements, and data are presented as means ± SD; two independent experiments were performed. *P < 0.05 and **P < 0.01, α-lactose-control, *Pb*ANKA-iRBC-stimulated cells, and *Pb*ANKA-iRBC-stimulated cells treated with α-lactose vs. PBS control group; ^#^*P* < 0.05, *Pb*ANKA-iRBC-stimulated cells treated with α-lactose vs. *Pb*ANKA-iRBC-stimulated cells.

We also investigated the expression of galectins, the receptors of Gal-9, and cytokines *in vitro*. Co-incubation of peritoneal macrophages with *Pb*ANKA-iRBCs *in vitro* significantly increased the mRNA levels of Gal-9, Tim-3, IFN-α, IFN-β, IFN-γ, and IL-10 (*P* < 0.05), but not Gal-1, Gal-3, Gal-8, CD44, CD137, or PDI (Fig. 8a–c). The increased expressions of Gal-9 and Tim-3 suggested that they were the major ligand-receptor pair responding to *Pb*ANKA-iRBCs. Treatment of the cultures with α-lactose significantly increased the levels of IFN-α and IFN-β (*P* < 0.05), but not IL-4, IL-10, or IFN-γ. Blockage of the Gal-9 and Tim-3 binding by α-lactose may not affect the expression of the two genes, but may influence their function in signaling leading to changes in expression of downstream genes. The increase in the levels of IFN-α and IFN-β in co-cultured peritoneal macrophages were consistent with those measured in the lung *in vivo* (Fig. 7), suggesting the involvement of these cytokines in inflammation during ALI. However, the mechanism of how the IFN-I influences ALI required further investigation.

## Discussion

Malaria-associated lung pathology is one of the frequent malaria complications that have not received enough attention^20^. Severe malaria is often caused by excessive immune responses, not due to an absence of immunity, particularly after T cell priming^21^. Inflammatory cellular infiltration, capillary permeability, or endothelial impairment leading to diffuse alveolar damage, alveolar edema, and focal alveolar hemorrhage are the major features of malaria-induced ALI/ARDS in humans^4^. Some features of lung injury in experimental severe malaria have been previously described, such as high parasite burden, leucocyte accumulation, CD36-mediated sequestration^5^, and increased expression of circulating vascular endothelial growth factor (VEGF)^22^. However, the immunoregulatory mechanisms of ALI during malaria are still poorly understood. In the present study, we successfully constituted a *Pb*ANKA-induced ALI in KM mice with dyspnea or respiratory insufficiency occurring between 5 to 7 days p.i. before death. Lung histopathological damage, characterized by inflammatory cellular infiltration, alveolar edema, and hemorrhage, were observed from day 5 p.i. There were also increased levels of BALF total protein, which is indicative of alveolar-capillary membrane barrier disruption^23^. However, malarial mice treated with α-lactose had lower survival rate and died between 5 and 7 days p.i. in comparison of died between 5 and 22 days p.i. in malarial mice. It has been reported that high numbers of inflammatory cells are observed in lung biopsies from patients and mice that succumbed from malaria associated ALI^4^, and the presence of edema, inflammatory cells, and infected erythrocytes is a hallmark of both human and experimental malaria-associated ARDS caused by different *Plasmodium* species^24^.

Galectins are potent immune regulators in a variety of pathological processes including inflammation, autoimmunity, fibrosis, and cancer^25^. In addition to its regulatory roles on T cells, Gal-9 can expand populations of immunosuppressive macrophages to ameliorate T-cell-mediated lung inflammation^26^. In our study, we measured the mRNA levels of Gal-1, Gal-3, Gal-8, and Gal-9 after infection with *Pb*ANKA. We found that Gal-9 was significantly upregulated in the lungs and MLNs, while Gal-3 and Gal-8 were only significantly increased in the MLNs of both malarial mice and malarial mice treated with α-lactose. Gal-3 is an endogenous modulator of inflammatory processes and anti-infective agents. Mice deficient in Gal-3 did not alter the course of parasitaemia during *P*. *berghei* infection but significantly reduced *P*. *yoelii* parasitaemia, indicating that endogenous Gal-3 could control experimental malaria in a species-specific manner^27^. Gal-8 is expressed abundantly in many tissues^28^ and is able to co-stimulate T cells^29^. Gal-9 acts as a ligand for Tim-3, CD44, CD137, and PDI^14^,^15^,^16^. Thus, the mRNA levels of Gal-9 receptors in the lungs and MLNs were measured in the present study. The results showed that Tim-3, CD44, CD137, and PDI were significantly increased in the lungs and/or MLNs of both malarial mice and/or malarial mice treated with α-lactose. Our data suggested that the signaling of Gal-9, together with its receptors (Tim-3, CD44, CD137, and PDI) play a role in the development of ALI in *Pb*ANKA-infected mouse model. Besides Tim-3, CD44 is another known cell surface molecule that can potentially interact with Gal-9^14^. CD44 binds not only to Gal-9 but also to its major ligand—hyaluronic acid (HA), a large glycosaminoglycan abundant in mammalian tissues. Gal-9 not only affects the Th1/Th2 balance mediated by Tim-3 but also involves in leukocyte migration into the inflammatory site. Gal-9 binds to CD44 and inhibits leukocyte migration during allergic lung inflammation via modulation of CD44−HA interactions^30^. Anti-Gal-9 Ab treatment promoted conjunctival eosinophil infiltration by increasing CD44−HA interaction^31^. Gal-9 can also bind to CD137 and PDI. CD137 is an activation-induced co-stimulatory molecule and an important regulator of immune responses^32^. Gal-9 directly binds to CD137, and facilitates CD137 aggregation, signaling, and functional activity in T cells, DCs, and NK cells^15^. PDIs are a family of redox chaperones that are implicated in protein unfolding and trafficking across the endoplasmic reticulum and towards the cytosol, a thiol-based redox locus for antigen processing^33^. PDI can assist protein folding in malaria parasites^34^. Gal-9 facilitates HIV entry into CD4^+^ T cells through PDI in a Tim-3-independent manner and thus could result in increased viral replication^35^. In the present study, significantly increased levels of CD44, CD137, and PDI were detected in the lungs and/or MLNs of malarial mice treated with α-lactose. These results suggest that Gal-9 interacts with multiple receptors, and all of these receptors likely play a role in the malaria-associated ALI in this mouse model.

Studying lung macrophages for different phenotypic markers, their polarization, activation, and recruitment may identify intervention measures for improving the outcome of ALI^36^. In the present study, we compared macrophage infiltration in the lungs between malarial mice with or without α-lactose treatment. Our results showed that compared with normal control, more CD68^+^-Gal-9^+^ and CD68^+^-Tim-3^+^ cells were observed in the lung tissues of α-lactose-control, malarial mice, and malarial mice treated with α-lactose; further, more infiltration of CD68^+^ macrophages were observed in the lung tissues of malarial mice treated with α-lactose than those in malarial mice on day 7 p.i. The data were consistent with that of the increased mRNA expressions of Gal-9 and Tim-3 in the lungs and MLNs of both malarial mice and malarial mice treated with α-lactose. Gal-9 is expressed in many cell types, including eosinophils, T cells, DCs, macrophages, lymphoid cells, and Kupffer cells^37^,^38^. Similarly, Tim-3 is expressed on a variety of immune cells, such as T cells, monocytes, macrophages, and DCs, and regulates the innate immune response^39^. Previous works from our laboratory have shown the upregulation of Tim-3 and Gal-9 in *Pb*ANKA-induced ALI in the mouse model^40^. In the current study, we further demonstrated that Gal-9 and Tim-3 were expressed mainly on infiltrating mononuclear cells in the lung tissues after *Pb*ANKA infection. Studies have reported the involvement of TIM-3/Gal-9 interaction in activation of neutrophils and macrophages^41^,^42^. In addition, CD44 has been shown to play a critical role in the recruitment of macrophages into the lung in response to inhaled Lipopolysaccharide (LPS)^42^,^43^ and in preventing exaggerated inflammatory responses to LPS by promoting the expression of the negative regulators of TLR4 signaling in macrophages^44^. The increased CD44 expression in *Pb*ANKA-infected mice suggests that CD44 may also contribute to the recruitment of macrophages into the lung during malaria-associated ALI, which requires further investigation. Similarly, Gal-9/CD137 and Gal-9/PDI interactions may also play a role during ALI.

Although the actual regulatory mechanisms for controlling the expressions of cell surface Gal-9 and Tim-3 were unknown, the increase of Gal-9^+^ and Tim-3^+^ mononuclear cells was likely a reason for enhancement of Gal-9 and Tim-3 expressions in *Pb*ANKA-induced ALI. Gal-9 attenuates pathology and levels of proinflammatory cytokines in LPS-induced ALI mice^45^. Our data suggest that Tim-3 and Gal-9 on mononuclear cells may play an important role in the development of ALI in murine malaria; more infiltration of mononuclear cells as well as other immune cells into the lungs increased the lung pathology during pulmonary *Pb*ANKA infection in malarial mice possibly due to blockage of galectines by α-lactose. It has been reported that T regulatory cells (Tregs), key mediators of immune homeostasis, are increased in number and modulate disease in human and murine malaria^46^. Gal-9 is highly expressed in induced Treg (iTreg) and was crucial for the generation and function of iTreg cells; Gal-9−CD44 interaction regulates iTreg differentiation and function. By utilizing Gal-9-deficient mice, it has been demonstrated that the genetic loss of Gal-9 leads to a reduction in Foxp3 expression and suppressor function of iTreg cells both *in vitro* and *in vivo*^14^. Thus, treatment of malarial mice with α-lactose may affect the functions of macrophages and T cells, particularly Treg cells, during ALI. Considering the critical role of iTreg cells in autoimmunity and tissue inflammation, altered activations and functions of macrophages and T cells may explain the more severe pathological changes, including increased extent of immune cell apoptosis, observed in the lungs of *Pb*ANKA-infected mice after α-lactose treatment.

Anti-inflammatory properties of IFN-I have received considerable attention recently. IFN-I are a family of cytokines prominent in antiviral responses consisting of multiple subclasses including IFN-α, IFN-β, IFN-ε, IFN-ω, and IFN-κ^47^. Studies have demonstrated that IFN-I can be induced in response to parasite infection such as *Plasmodium*, *T*. *gondii*, *Cryptosporidium*, *Trypanosomes*, and *Leishmania* and can influence the outcome of infection^48^. IFN-I mediate susceptibility to *Pb*ANKA using a murine model of severe malaria^49^. In the present study, we showed significantly increased levels of IFN-α, IFN-β, and IFN-γ in the lungs of malarial mice treated with α-lactose on day 5 p.i. in comparison of malarial mice. However, significantly reduced levels of IFN-α and IFN-β in the lungs and MLNs on day 7 p.i. in malarial mice after α-lactose treatment could be partly due to the death of immune cells such as macrophages, lymphocytes, and neutrophils. IFN-α and IFN-β can induce excessive inflammation^50^. Anti-Gal-9 Ab treatment has been shown to significantly reduce IL-5 and IL-13 production while significantly elevates IFN-γ production of murine splenocytes^31^. It was also found that Gal-9 was induced by IFN-γ in inflamed tissue during Th1 cell responses and in turn induced apoptosis of Th1 cells via Tim-3 and thus could control inflammation^39^. However, in the current study, quantitative analysis of TUNEL-positive lung cells showed that the number of apoptotic cells in the lungs of malarial mice with blockage of galectins by α-lactose was significantly higher than those in the malarial mice. Blockage of galectins could lead to apoptosis of iTreg cells, elevated levels of cytokines such as IFN-I, higher inflammatory response, death of immune cells, and tissue damages in the lung during ALI. It has been reported that IFN-α, IFN-β, and IFN-γ induce apoptosis in multiple cell lines of varied histologies *in vitro*^51^. IFN-α and IFN-β have also been associated with necroptosis; IFN-I-dependent induction of macrophage necroptosis is a major mechanism by which the pathogen deactivates the innate immune responses in mice infected with *S*. Typhimurium^52^. Our data indicated that blockage of galectins with α-lactose may alter the pathogenic properties of *Pb*ANKA-inducing immune cells and increase apoptotic cells in malarial mice treated with α-lactose that could be mediated by increased production of IFN-I.

In addition, compared with malarial group, there were significantly increased levels of IL-4 and IL-10 in the lungs and significantly increased IL-10 in the MLNs of malarial mice treated with α-lactose in this study. It has been reported that Tim-3 blockage *in vivo* increased IL-10 expression in a mouse sepsis model^53^. Blockage of Tim-3 signaling in monocytes also results in increased IL-10 production^54^. IL-10 was also associated with high levels of parasitemia of *P*. *falciparum* in patients with CM and non-CM^55^. High IL-10 levels have been linked to a decreased ability to eliminate malaria parasitemia in children in Tanzania^56^. IL-10 is known as an anti-inflammatory cytokine by many cell types, including monocytes and, to a lesser extent, by lymphocytes such as Th2 cells, mast cells, CD4^+^CD25^+^Foxp3^+^ Tregs, and in a certain subset of activated T cells and B cells^57^. Its expression is often associated with increased inflammation (e.g., to suppress inflammatory response), and it has been suggested that IL-10 may be used as a biomarker for inflammatory placental malaria^58^. In this study, it was likely that high level of IL-10 was produced after damages were done, e.g. after or about the same time when inflammatory cytokines were produced. Thus, upregulation of IL-10 expression after blockage of galectins *in vivo* in our study can be considered as host response to inflammation.

In summary, we show that malaria infection causes ALI that is likely due to elevated/excessive inflammatory responses mediated by increased immune cell infiltration and cytokine production, particularly IFN-I and IFN-II. Gal-9 plays an important role in this process through interactions with its receptors including Tim-3, CD44, CD137, and PDI; blockage of their interactions using α-lactose enhances inflammatory response and exacerbates ALI and tissue damage. A better understanding the molecular mechanism of severe malaria may provide crucial information to alleviate the suffering from this deadly disease.

## Materials and Methods

### Ethics Statement

KM mice (outbred) were obtained from the Animal Center of Sun Yat-sen University, maintained in specific-pathogen-free environment, and had free access to a commercial basal diet and tap water *ad libtum*. Animals were provided with humane care and healthful conditions during their stay in the facility. All individuals who used animals received instruction in experimental methods and in the care, maintenance, and handling of mice; and all efforts were made to minimize animal suffering. Animals were sacrificed using CO_2_ asphyxiation and the appropriate organs were harvested. The protocol in this study was approved by the Committee on the Ethics of Animal Experiments of the Sun Yat-sen University [Permit Number: SCXK (Guangdong) 2009–0011], and all the experiments were performed in accordance with relevant guidelines and regulations.

### Mice, treatment with α-lactose, and experimental infections

Female KM mice (6–8 weeks old) and *Pb*ANKA were used throughout the study. Some mice were injected intraperitoneally (i.p.) with 300 mM of α-lactose solution in PBS twice daily starting from day 1 post infection (p.i.) until the day mice were sacrificed. Animals were sacrificed and their organs were taken for further analysis 12 hrs after the last treatment^59^. A total of 65 mice were used in the experiments: 33 mice were injected i.p. with 10^6^ *Pb*ANKA-iRBCs; 23 mice were injected i.p. with 10^6^ *Pb*ANKA-iRBCs and treated with α-lactose; 4 mice were injected i.p. with α-lactose alone; and 4 mice were injected with equal volume of phosphate buffered saline (PBS) as negative controls. Control animals received an equivalent volume of diluted normal red blood cells. Mortality was monitored daily. Four *Pb*ANKA-infected mice were sacrificed by CO_2_ asphyxiation for examination on days 5 and 7 p.i., respectively. Parasitemia was monitored daily by Giemsa-stained thin blood smears of tail blood. Erythrocyte counts were performed with a hematocytometer, and more than 1,000 RBCs were counted by microscopy (×100) to determine the percentage of parasitized RBCs.

### BALF analysis

BALFs were obtained by instillation and aspiration of 0.6 ml aliquots of PBS from *Pb*ANKA-infected mice with or without α-lactose treatment on day 7 p.i.; *Pb*ANKA-infected mice, uninfected control mice, and mice treated with α-lactose alone were sacrificed at the same time. The BALFs were spun at 800 g at 4 °C for 5 min, and the supernatants were stored at −80 °C for further analysis. Total protein concentrations of the BALFs were measured using a BCA protein assay (Sigma-Aldrich).

### Histopathology

For histopathological analysis, the lungs and MLNs from *Pb*ANKA-infected, *Pb*ANKA-infected with α-lactose treatment, or control mice were harvested and immediately fixed in 10% buffered formaldehyde (Guangzhou Chemical Reagent Factory, China) for 48 h. Five-micrometer-thick sections (50- or 100-μm distance between sections) of the organs from mice were stained with hematoxylin and eosin (H&E) (Sigma-Aldrich) and evaluated for histological changes. Sections, blinded for groups, were analyzed by a pathologist. To score lung inflammation and damage, a semi-quantitative score described previously^60^ was used. Briefly, the following parameters were analyzed: interstitial inflammation, intra-alveolar inflammation, edema, endothelialitis, bronchitis, pleuritis, and thrombi formation. Each parameter was graded on a scale from 0 to 3 as follows: 0, absent; 1, mild; 2, moderate; and 3, severe. The total lung inflammation score was expressed as the sum of the scores for each parameter; the maximum being 21.

### Immuno-staining for CD68 in the lungs and MLNs and morphometric analysis

Immunohistochemistry was carried out using the streptavidin–biotin–peroxidase complex (SABC) method. Tissue sections (5-μm) were deparaffinized and rehydrated in distilled water. Heat-induced antigen retrieval was carried out in an 800-W microwave oven for 30 min. Endogenous peroxidase activity was blocked by incubation with 3% hydrogen peroxide in methanol for 10 min at 37 °C. Nonspecific binding was blocked by incubation in 5% bovine serum albumin (Sigma-Aldrich) in PBS (pH 7.4) for 10 min at room temperature. The sections were incubated with rabbit anti-CD68 (Wuhan Boster Biological Engineering Co., Ltd., Wuhan, China) (1:200 dilutions) overnight at 4 °C, and then secondary antibodies. Sections incubated with secondary antibodies only were used as isotype controls. Signals were detected with a SABC kit and developed in diaminobenzidine tetrahydrochloride (Zhongshan Golden Bridge Technology, Beijing, China). The sections were counterstained with hematoxylin and examined under a light microscope. CD68-positive cells were identified by dark-brown staining.

CD68-positive cells were quantified using images captured with a digital camera system and analyzed by using Image-Pro Plus (Image Z1 software, Media Cybernetics, MD, US). The number of cells in each field was determined under high power field as well as the area of each field (0.015066 mm^2^). The density of positive cells was expressed as the number of cells per square millimeter.

### Immunofluorescence assay

BALFs of the mice infected with *Pb*ANKA and uninfected controls were spun at 800 g at 4 °C for 5 min, and the pelleted cells were resuspended and seeded on glass coverslips in 24-well plates (Corning, NY, USA). After 4 h at 37 °C in a 5% CO_2_ atmosphere, cells were washed and cultured in Dulbecco’s modified Eagle’s medium (DMEM) (Sigma-Aldrich) with 10% FBS (Gibco, NY, USA). The coverslips with adherent cells from the BALFs were fixed with 4% paraformaldehyde in 0.1 M phosphate buffer for 15 min, rinsed twice with PBS, and then used for double immunofluorescence labeling. The cells were incubated with DAPI (1:50,000; Sigma-Aldrich) plus mouse anti-mouse CD-68 monoclonal antibody (IgG1; 1:200; Abcam, Cambridge, UK) and rabbit anti-Tim-3 polyclonal antibody (IgG1; 1:200 dilution; Abcam) or rabbit anti-Gal-9 monoclonal antibody (IgG1; 1:200 dilution; Bioss, Beijing, China) overnight at 4 °C. Subsequently, the cells were incubated with anti-mouse IgG (H + L), F (ab’)_2_ fragment (Alexa Fluor^®^ 488 Conjugate) (2 mg/ml, 1:200 dilution; Cell Signaling Technology, MD, USA) and anti-rabbit IgG (H + L), F (ab’)_2_ fragment (Alexa Fluor^®^ 555) (2 mg/ml, 1:200 dilution; Cell Signaling Technology) together for 60 min at room temperature in a dark chamber. The slides were washed three times with PBS (pH 7.4) for 30 min at room temperature and mounted with antifade polyvinylpyrrolidone mounting medium (Beyotime, Haimen, China) in a dark chamber. For negative controls, a set of culture slides was incubated under similar conditions without the primary antibodies. CD68^+^–macrophages were identified by their green fluorescent, whereas Tim-3 and Gal-9 appeared as red fluorescent located in the nucleus or cytoplasm, once CD68 and Gal-9 or CD68 and Tim-3 superimposed in one image that would appear as yellow fluorescent. Two pathologists observed the sections under a fluorescence microscope (BX63, Olympus, Japan).

### Apoptosis assay

Signals of apoptosis in 5-μm lung paraffin sections were detected using TUNEL assay (Wuhan Boster Biological Engineering Co., Ltd.). Negative control was prepared by omission of terminal deoxynucleotidyl transferase. TUNEL-positive cells were determined under high power field as well as the area of each field (0.015066 mm^2^). The density of positive cells was expressed as the number of cells per square millimeter.

### Isolation and culture of murine peritoneal macrophages

KM mice were injected intraperitoneally (i.p.) with 2 ml 3% thioglycollate broth (Sigma-Aldrich) solution in PBS once daily for 3 days. Animals were sacrificed and their peritoneal lavage fluid were spun at 800 g at 4 °C for 5 min, and the pelleted peritoneal macrophages were resuspended and seeded at 5 × 10^5 ^cells/well in 12-well plates (Corning, NY, USA). After 4 h at 37 °C in a 5% CO_2_ atmosphere, cells were washed and cultured in Dulbecco’s modified Eagle’s medium (DMEM) (Sigma-Aldrich) with 10% FBS (Gibco, NY, USA). Macrophages were co-cultured with 5 × 10^6^ *Pb*ANKA-iRBCs for 24 h. To block the binding of autocrine Gal-9 and its receptors, cells were pre-incubated with 30 mM α-lactose for 1 h before adding *Pb*ANKA-iRBCs. Samples were stored at −80 °C until subjected to further analysis.

### Measurement of mRNA expression using quantitative real-time PCR (qRT-PCR)

Total RNA was extracted from about 100 mg of mouse lung and MLN tissues or macrophages of each group using a RNA Extraction Kit (TaKaRa Bio, Inc., Tokyo, Japan) according to the manufacturer’s protocol. The quality of total RNA was analyzed by running 5 μl of each RNA sample on a 1.0% agarose gel stained with ethidium bromide. The quantity of total RNA was estimated by measuring the ratio of absorbance at 260 and 280 nm using a NanoDrop ND-1000 spectrophotometer (NanoDrop Technologies, DE, USA). First-strand cDNA was constructed from 1.0 μg of total RNA with oligo (dT) as primers using a PrimeScript 1^st^ Strand cDNA Synthesis Kit (TaKaRa Bio, Inc.) following the manufacturer’s protocol. cDNA was stored at −80 °C until use. To determine tissue or macrophage mRNA levels of Gal-1, Gal-3, Gal-8, and Gal-9, Tim-3, CD44, CD137, PDI, and IFN-α, IFN-β, IFN-γ, IL-4, and IL-10, qRT-PCR was performed using SYBR Green QPCR Master Mix (TaKaRa Bio, Inc.) according to the manufacturer’s instructions. For lung parasite burden, mRNA level of *Pb*ANKA 18S rRNA measured by qRT-PCR is shown as −∆∆Ct values. Primers for the qRT-PCR are listed in Supplemental Table 1. Briefly, a total of 10 μl reaction mixture contained 5.0 μl of SYBR^®^ Premix Ex TaqTM (2×), 0.5 μl of each primer (10 pM), 3.0 μl of dH_2_O, and 1.0 μl of cDNA (0.2 μg/μl). Amplification was pre-denaturized for 30 s at 95 °C followed by 43 cycles of 5 s at 95 °C and 20 s at 60 °C with a LightCycler^®^ 480 instrument (Roche Diagnostics, AL USA). Specific mRNA expression levels were normalized to that of the housekeeping gene, β-actin, and the results are expressed as fold change compared to uninfected controls.

### Statistical analysis

Results of experimental studies were reported as mean ± SD. Statistical analysis of the data was performed using Wilcoxon rank sum test, Student’s t test, and one way ANOVA followed by Bonferoni’s multiple comparison tests using SPSS software for windows (version 19.0; SPSS, Inc., IL, USA). All graphs were performed using GraphPad Prism software and a value of *P* < 0.05 was considered statistically significant.

## Additional Information

**How to cite this article**: Liu, J. *et al*. Blockage of Galectin-receptor Interactions by α-lactose Exacerbates *Plasmodium berghei*-induced Pulmonary Immunopathology. *Sci. Rep.* **6**, 32024; doi: 10.1038/srep32024 (2016).

## Supplementary Material

Supplementary Information
