# Supplementary material for: Blockage of Galectin-receptor Interactions by α-lactose Exacerbates Plasmodium berghei-induced Pulmonary Immunopathology
Source: Sci Rep. 2016 Aug 24;6:32024. doi: 10.1038/srep32024 (PMC4995515; doi:10.1038/srep32024)
Supplement: Supplementary Information [file srep32024-s1.pdf]

Blockage of Galectin-receptor Interactions by α-lactose Exacerbates *Plasmodium berghei*-induced Pulmonary Immunopathology

Jinfeng Liu[1, 2], Shiguang Huang[3], Xin-zhuan Su[4, 5]*, Jianping Song[6], Fangli Lu[1, 2]*

[1] Department of Parasitology, Zhongshan School of Medicine, Sun Yat-sen University, Guangzhou 510080, Guangdong, China

[2] Key Laboratory of Tropical Disease Control (Sun Yat-sen University), Ministry of Education, Guangzhou 510080, Guangdong, China

[3] School of Medicine, Jinan University, Guangzhou 510632, Guangdong, China

[4] Laboratory of Malaria and Vector Research, National Institute of Allergy and Infectious Diseases, National Institutes of Health, Bethesda, Maryland 20892, United States of America

[5] State Key Laboratory of Cellular Stress Biology, Innovation Center for Cell Signaling Network, School of Life Sciences, Xiamen University, Xiamen, Fujian 361005, China

[6] Institute of Science and Technology, Guangzhou University of Chinese Medicine, 436 Chentai Road, Baiyun District, Guangzhou 510445, Guangdong, China

Running head: Blockage of Galectins Exacerbates Pulmonary Immunopathology

*Corresponding authors:

X.-z.S; Email: xsu@niaid.nih.gov

F. L.; Email: fanglilu@yahoo.com

Supplemental Table 1. Primer sequences of mouse target cytokines and housekeeping genes used for quantitative real-time polymerase chain reaction (qRT-PCR) assays

Genes	Primer sequence (5′→3′)	Accession
*Pb*ANKA 18S	Forward primer AAGCATTAAATAAAGCGAATACATCCTTAC	NW_672388.1
	Reverse primer GGAGATTGGTTTTGACGTTTATGTG	
IFN-α	Forward primer CTTTGGATTCCCGCAGGA	NM_010502.2
	Reverse primer TGTAGGACAGGGATGGCTTGA	
IFN-β	Forward primer TGAATGGAAAGATCAACCTCACCTA	NM_010510.1
	Reverse primer CTCTTCTGCATCTTCTCCGTCA	
IFN-γ	Forward primer GGAACTGGCAAAAGGATGGTGAC	NM_008337.4
	Reverse primer GCTGGACCTGTGGGTTGTTGAC	
IL-10	Forward primer AGCCGGGAAGACAATAACTG	NM_010548.2
	Reverse primer CATTTCCGATAAGGCTTGG	
	Reverse primer CATTTCCGATAAGGCTTGG	
IL-4	Forward primer ACAGGAGAAGGGACGCCAT	NM_021283.2
	Reverse primer GAAGCCCTACAGACGAGCTCA	
Gal-1	Forward primer CGCCAGCAACCTGAATC	NM_008495.2
	Reverse primer GTCCCATCTTCCTTGGTGTTA	
Gal-3	Forward primer GCTACTGGCCCCTTTGGT	NM_001145953.1
	Reverse primer CCAGGCAAGGGCATATCGTA	
Gal-8	Forward primer GGGTGGTGGGTGGAACTG	NM_001199043.1
	Reverse primer GCCTTTGAGCCCCCAATATC	

Gal-9	Forward primer GTTGTCCGAAACACTCAGAT	NM_001159301.1
	Reverse primer ATATGATCCACACCGAGAAG	
Tim-3	Forward primer CCACGGAGAGAAATGGTTC	NM_134250.2
	Reverse primer CATCAGCCCATGTGGAAAT	
CD44	Forward primer TGCAGGTATGGGTTCATAGAAGG	NM_001039150.1
	Reverse primer GTGTTGGACGTGACGAGGA	
CD137	Forward primer CGTGCAGAACTCCTGTGATAAC	NM_001077508.1
	Reverse primer GTCCACCTATGCTGGAGAAGG	
PDI	Forward primer CGCCTCCGATGTGTTGGAA	NM_007952.2
	Reverse primer GAAGAACTCGACTAGCATGAGC	
β-actin	Forward primer TGGAATCCTGTGGCATCCATGAAAC	NM_007393.5
	Reverse primer TAAAACGCAGCTCAGTAACAGTCCG	
